# Notes on the Use of Antiseptics in Surgery with Special Reference to the Pine Product Called Snitas

**Published:** 1888-07

**Authors:** Frederick Bowreman Jesset

**Affiliations:** Surgeon to the Cancer Hospital, London


### NOTES ON THE USE OF ANTISEPTICS IN SURGERY WITH SPECIAL REFERENCE TO THE PINE PRODUCT CALLED SNITAS.

BY FREDERICK BOWREMAN JESSET, F. R. C. S.
Surgeon to the Cancer Hospital, London.

For some considerable time I have been in search of a substance for use during operations, and for dressing wounds after operations, which shall in itself meet all the requirements of antiseptic surgery. It appeared to me that in searching for such a substance, out of the long list of antiseptics, I should be guided, to a very great extent at any rate, in my selection, by the following requirements, viz:

I. The substance must be proved to possess the power of preventing organic matters from passing intoa state of putrefaction, and at the same time destroy any poisonous products whice are generated thereby.

2. It should be non-pooisonous and non-irritating.

3. It should not injuriously affect by any chemical action surgical instruments that are brought into contact with it.

4. Its composition should be of the simplest, and the price moderate. to meet the exigencies of hospital practice.

(The writer tried carbolic bichloride, iodoform and salicylic acid but found them all unsatisfactory.)

It appeared to me, then, that if I could find a substance which would combine in itself all the antiseptic properties of the above named substances in an equal degree, and at the same time be applicable for use both during thc performance of an operation and as a dressing afterwards, be non-irritating, perfectly inocuous, volatile, not likely to deteriorate through changes of atmosphere, I should attain everything that could be required.

Such an antiseptic, I have no hesitation in saying, I believe I have found a substance named sanitas, which is one of the pine products, and consists essentially of peroxide of hvdrogen, camphoric acid, camphor, thymol, and an oxidized oil containing a large quantity of camphoric peroxide.

By experiments, I fully convinced myself as to the power of sanitas to prevent fermentation and putrefaction, and to destroy any disease germs or sep.ic matter with which it came into contact, it, unlike carbolic acid and corrosive sublimate—which is a great promoter of the process of oxidation, and, being non-poisonous, is an agreable and natural antiseptic, and has the further advantage of leaving no stain whatever on the most delicate fabrics.

Here, then, is a substance which meets every requirement I had looked for, viz: A non-irritating, innocuous, volatile substance, freely soluble in water, and therefore aplicable for use during operations; and for dressing after operations; it is everything that could be desired, while from its volatile nature the atmosphere is impregnated with a pleasant, invigerating odor.

From a now considerable experience of the use of sanitas as a dressing, I am able to report most highly of its antiseptic qualities in the following class of cases, viz:

1, In all cases of operation.
2. As a dressing in cases of putrid, foul ulcers.
3. As an injection in ulceration or malignant disease of the uterus.
4. In a concentrated form (equal parts of sanitas, oil and glycerin) for saturating tampons of absorbent wool for plugging the vagina in very bad cases of cancer of the uteaus.
5. As a mouth-wash in cancer of the mouth and tongue.

—*Epitome.*

